# The steroid hormone 20-hydroxyecdysone inhibits RAPTOR expression by repressing *Hox* gene transcription to induce autophagy

**DOI:** 10.1016/j.jbc.2024.108093

**Published:** 2024-12-18

**Authors:** Tian-Wen Liu, Can Tian, Yan-Xue Li, Jin-Xing Wang, Xiao-Fan Zhao

**Affiliations:** Shandong Provincial Key Laboratory of Animal Cells and Developmental Biology, School of Life Sciences, Shandong University, Qingdao, China

**Keywords:** RAPTOR, 20-hydroxyecdysone, homeobox, cell proliferation, autophagy

## Abstract

Regulatory-associated protein of TOR (RAPTOR) is a key component of TOR complex 1, which determines the lysosomal location and substrate recruitment of TOR complex 1 to promote cell growth and prevent autophagy. Many studies in recent decades have focused on the post-translational modification of RAPTOR; however, little is known about the transcriptional regulatory mechanism of *Raptor.* Using the lepidopteran insect cotton bollworm (*Helicoverpa armigera*) as model, we reveal the transcriptional regulatory mechanism of *Raptor*. RAPTOR has different expression profiles in tissues during development from larva to late pupa, with high expression levels at larval feeding stages but low expression levels during metamorphic stages in the epidermis, midgut, and fat body. RAPTOR is localized in the larval midgut at the feeding stage but is localized in the imaginal midgut at metamorphic stages. The knockdown of *Raptor* at the feeding stage results in the production of small pupae, early autophagy of the midgut and fat body, and decreased cell proliferation. However, *Raptor* knockdown at metamorphic stage represses the development of the epidermis, adult fat body, and brain. 20-Hydroxecdysone (20E) represses *Raptor* transcription. Homeobox (HOX) proteins promote *Raptor* transcription by binding to its promoter. Overexpression of HOX proteins represses autophagy-related gene expression and autophagy but increases cell proliferation. 20E represses *Hox* genes transcription *via* its nuclear receptor EcR binding to its promoters. Together, these findings suggest that HOX proteins are positive regulators that upregulate *Raptor* transcription. 20E represses *Hox* gene transcription, thus repressing *Raptor* expression, resulting in autophagy and repressing cell proliferation during metamorphosis.

Mammalian target of rapamycin complex 1 (mTORC1) promotes cell growth and proliferation by phosphorylating substrates such as ribosomal protein S6 kinase (S6K) and elF4E-binding protein 1 to promote protein and lipid synthesis or inhibits autophagy by phosphorylating unc-51-like autophagy-activating kinase 1 (ULK1, ATG1 in yeast) (1). The regulatory-associated protein of TOR (RAPTOR) is critical for the assembly, lysosomal membrane location (2), stability, and substrate recruitment of mTORC1 (3). Under nutrient-rich conditions, RAPTOR anchors mTORC1 on the lysosomal membrane, and ULK1 interacts with it and is phosphorylated by TOR, thereby localizing the ULK1 complex to the lysosomal membrane and inhibiting the initiation of autophagy (4, 5). RAPTOR plays important roles in a variety of physiological processes, and the loss of *Raptor* leads to autophagy and neurodegeneration, such as maintaining the normal structure of the nervous system (6). In *Drosophila melanogaster*, the specific knockout of *Raptor* in muscle tissue causes the failure of emergence and a short lifespan (7). The expression of *Daf-15*, the *Raptor* in *Cryptorhabditis elegans*, is decreased, resulting in autophagy under limited nutritional conditions (8). Knockdown of *Raptor* or *Tor* caused earlier autophagy in the midgut and fat body in *D. melanogaster* (9). RAPTOR is also a treatment target for neurological disease; for example, *Raptor* downregulation rescues neuronal phenotypes in mouse models of the tuberous sclerosis complex (10). There have been many studies on post-translational modifications of RAPTOR; for example, RAPTOR is phosphorylated by AMP-activated protein kinase to lose its role in mTORC1 (11), whereas little is known about the transcription factors that upregulate or downregulate *Raptor* transcription and the related mechanisms.

*Homeobox* (*Hox*) genes are transcription factors that were originally discovered in *Drosophila* (12). These genes are responsible for the differentiation of somatic segments, organ formation, and regulation of functions in these areas (13). *Hox* genes in mammals are distributed along the anterior and posterior body axes of the organism and are important for embryonic development (14). HOX proteins regulate gene transcription by binding to specific DNA sequences of target genes (15). In mammals, most *Hox* genes are involved in promoting breast cancer development (16). Estrogen plays a regulatory role in this process (17). Estrogen indirectly promotes HOXC10 expression through its receptor (18). Different studies have shown that estrogen inhibits HOXB7 expression through its receptor estrogen receptor alpha (ERα) (19). These findings suggest that there is a close association between *Hox* genes and estrogen. A study in *Drosophila* revealed a new function for *Hox* genes that inhibit developmental autophagy (20). *Hox* genes are expressed at low levels during metamorphosis, and *Hox* overexpression is sufficient to prevent autophagy. These findings suggest that *Hox* genes are central regulators of autophagy (21). In addition, HOXC9 has been reported to inhibit autophagy in mammals (22). However, little is known about the mechanism by which *Hox* represses autophagy.

The insects that undergo complete metamorphosis, such as *Helicoverpa armigera*, have a lifespan of four developmental stages: egg, larva, pupa, and adult, with different morphological transitions initiated by the steroid hormone 20-hydroxecdysone (20E) (23). 20E is transformed from the release of ecdysone (E), which is synthesized in the prothoracic gland and released into tissues and hydroxylated at the 20-position carbon atom (24). 20E titer increases during metamorphosis (25). Midgut and fat body tissues undergo remodeling during the metamorphosis, with larval tissues degradation by autophagy and apoptosis and new adult tissues formation by proliferation from imaginal discs (26). The color of midgut changes from yellow to red, and the fat body is degraded into single cells. These phenomena indicate the occurrence of autophagy and apoptosis of larval tissues in *H. armigera* (27, 28). EcR, the nuclear receptor of 20E (29, 30), binds to 20E and forms a heterotrimeric transcription complex with ultraspiracle isoform (USP) to promote target gene expression through binding to an EcR binding element (EcRE) in the promoter regions of target genes to initiate insect metamorphosis (31). 20E promotes autophagy during metamorphosis by upregulating the expression of autophagy-related genes (ATGs) through the binding of EcR to the EcRE on the promoter of target genes (32), but little is known about how 20E promotes autophagy by inhibiting the transcription of genes involved in cell proliferation *via* the EcR. *H. armigera* is a good model for the study of autophagy because of its distinctive morphology in various stages, rapid growth, and ease of feeding.

In this study, we used the lepidopteran insect *H. armigera*, the cotton bollworm, as a model to investigate these questions. We found that RAPTOR promotes cell proliferation and prevents autophagy. The transcription factor HOX promotes the transcription of *Raptor* by binding to specific sequences in its promoter. However, 20E inhibits the transcription of the *Hox* gene by binding to the *Hox* promoter through its nuclear receptor EcR, which leads to a decrease in *Raptor* expression and causes autophagy in larval tissues.

## Results

### Bioinformatic analysis of RAPTOR

We identified one RAPTOR in the *H. armigera* genome, and the phylogenetic tree constructed with other species revealed that the RAPTOR in *H. armigera* clustered with that in *Bombyx mori* (Fig. S1*A*). Amino acid sequence alignment of RAPTOR in *H. armigera* with those in humans and mice showed that their amino acid sequences were highly similar to each other (Fig. S2*B*). We analyzed the structural domains of RAPTOR in various species and found that, similar to those in other species, RAPTOR in *H. armigera* had the same conserved N-terminal Raptor structural domain and repetitive WD40 domain (Fig. S2). Conserved structure of RAPTOR suggests that its function is conserved across animals.

### Expression profiles of RAPTOR in different tissues

**Figure 1:**
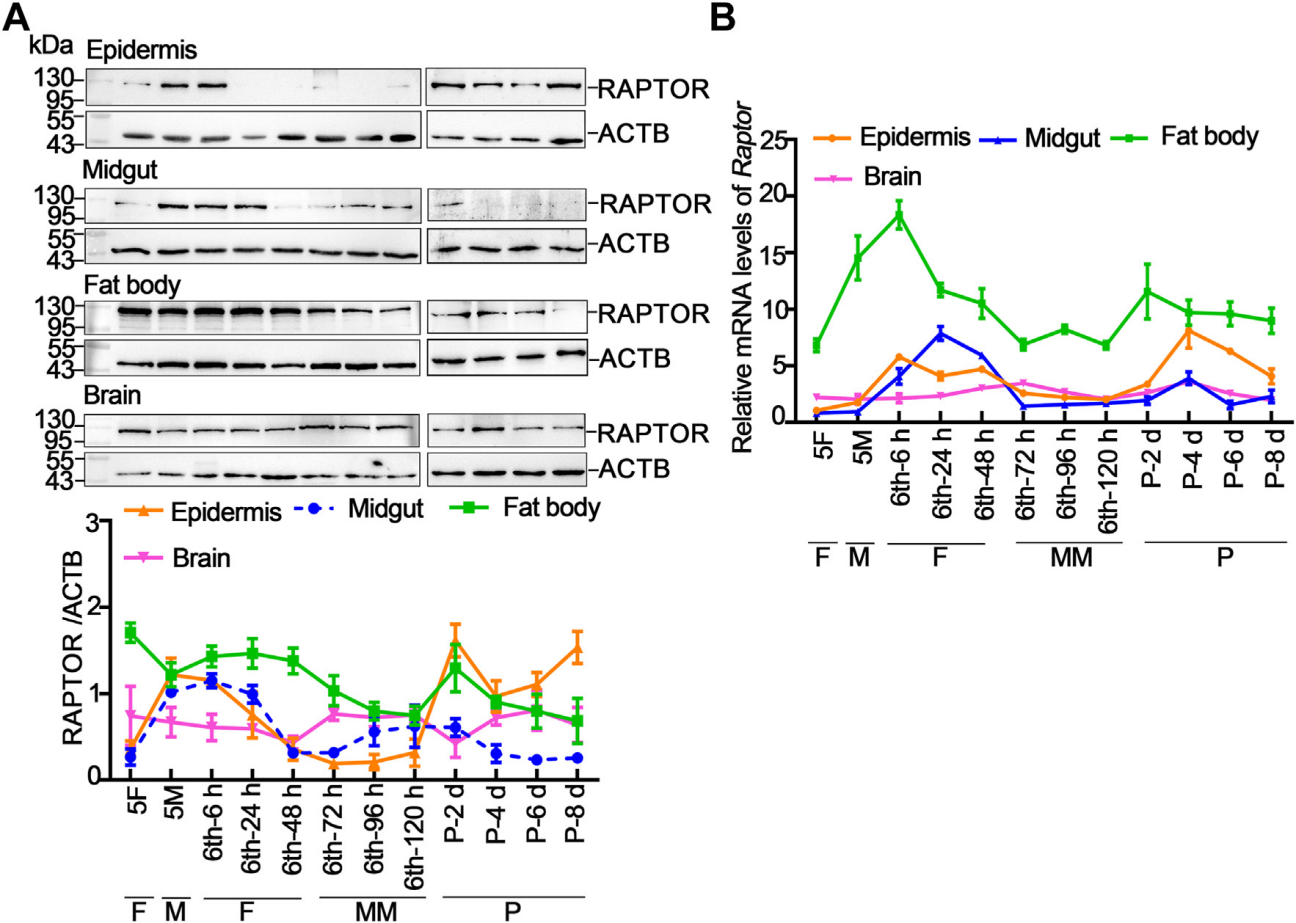
**RAPTOR expression profiles in different stages.***A*, protein expression levels of RAPTOR in the epidermis, midgut, fat body, and brain. ACTB was used as a protein quantity control. *B*, quantitative RT–PCR results showing the mRNA levels of *Raptor* at different stages. Actin was used as gene control. Statistical analyses of Figure 1, *A* and *B* are presented in Tables S1 and S2. F, feeding stage; 5F, fifth instar feeding larvae; M, molting stage; 5M, fifth instar molting larvae; MM, metamorphic molting stage; 6th-6 h to 6th-6 h–120 h: sixth instar 6 h to 120 h larvae; P, pupal stage; P-2 d to P-8 d, pupal period from day 2 to day 8; RAPTOR, regulatory-associated protein of TOR.

To investigate the function of RAPTOR in larvae, its expression profiles in the epidermis, midgut, fat body, and brain were examined using Western blotting. The results revealed that the expression profiles of RAPTOR differed across tissues during development from the larval to late pupal stages, with high expression levels at the larval feeding stages but low expression levels during metamorphic stages in the epidermis, midgut, and fat body, whereas the expression levels of RAPTOR were relatively even in the brain (Fig. 1*A*). Moreover, quantitative RT–PCR (qRT–PCR) showed that the mRNA expression level of *Raptor* was essentially consistent with the protein expression profile (Fig. 1*B*). These data indicate that RAPTOR expression levels are regulated in tissues at different developmental stages.

**Figure 2:**
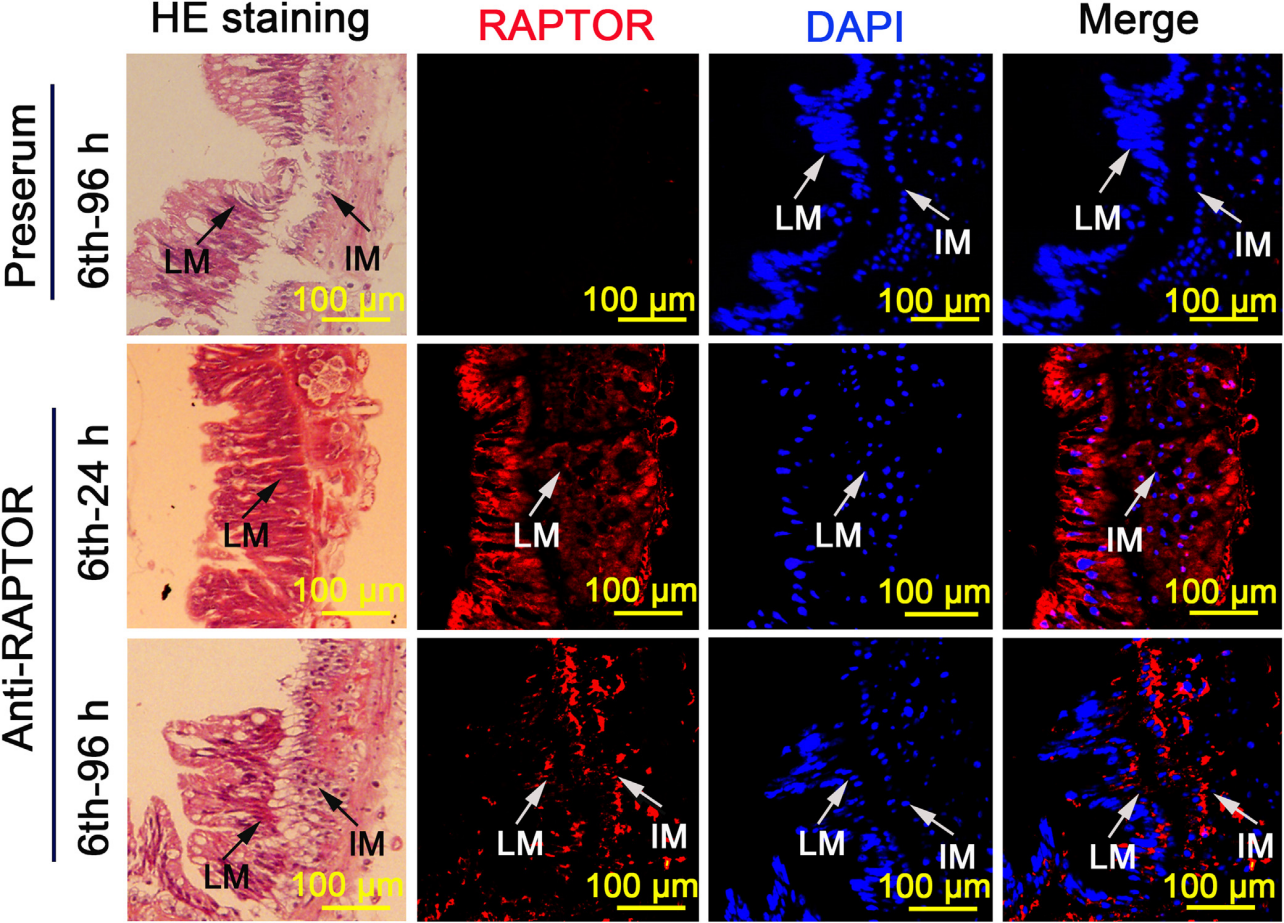
**Reduced distribution of RAPTOR in the larval midgut during metamorphosis.** H&E staining showing the morphology of the midgut. The location of RAPTOR was detected using immunohistochemistry. *Red*, RAPTOR detected using anti-RAPTOR antibodies; *blue*, nuclei stained with DAPI. DAPI, 4′,6-diamidino-2-phenylindole; IM, imaginal midgut; RAPTOR, regulatory-associated protein of TOR.

**Figure 3:**
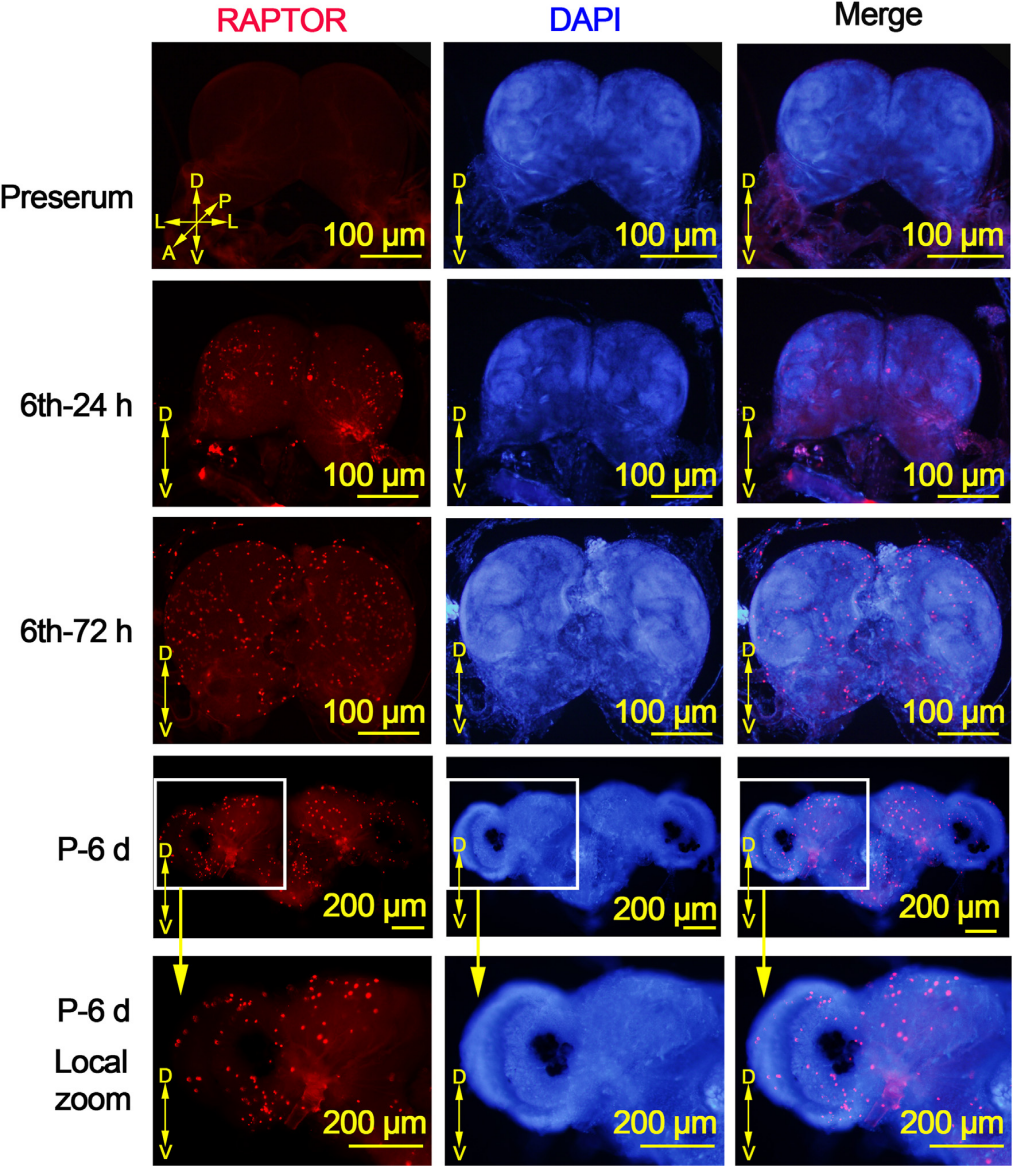
**Distribution of RAPTOR in the brain.** Localization of RAPTOR was detected by integral immunohistochemistry. *Red*, RAPTOR detected using anti-RAPTOR antibodies and goat antirabbit IgG secondary antibody DyLight594; *blue*, nuclei stained using DAPI. A, anterior; D, dorsal; L, lateral; P, posterior; V, ventral. DAPI, 4′,6-diamidino-2-phenylindol; RAPTOR, regulatory-associated protein of TOR.

To examine the variation in RAPTOR expression in the midgut during feeding and metamorphosis, we performed immunohistochemistry to observe its localization in the midgut at different ages. RAPTOR was largely localized in the midgut of larvae at 6th instar 24 h (6th-24 h). However, the distribution of RAPTOR in the larval midgut decreased in 6th–96 h larvae, and some RAPTOR was distributed in the imaginal midgut (Fig. 2). This suggests a possible role for RAPTOR in both larval and imaginal tissues. In contrast, RAPTOR was widely distributed in the brain from the larval stage to the pupal stage (Fig. 3). This indicates that RAPTOR also plays an important role in brain during development.

### RAPTOR promoted cell proliferation and prevented autophagy in the larval midgut and fat body

**Figure 4:**
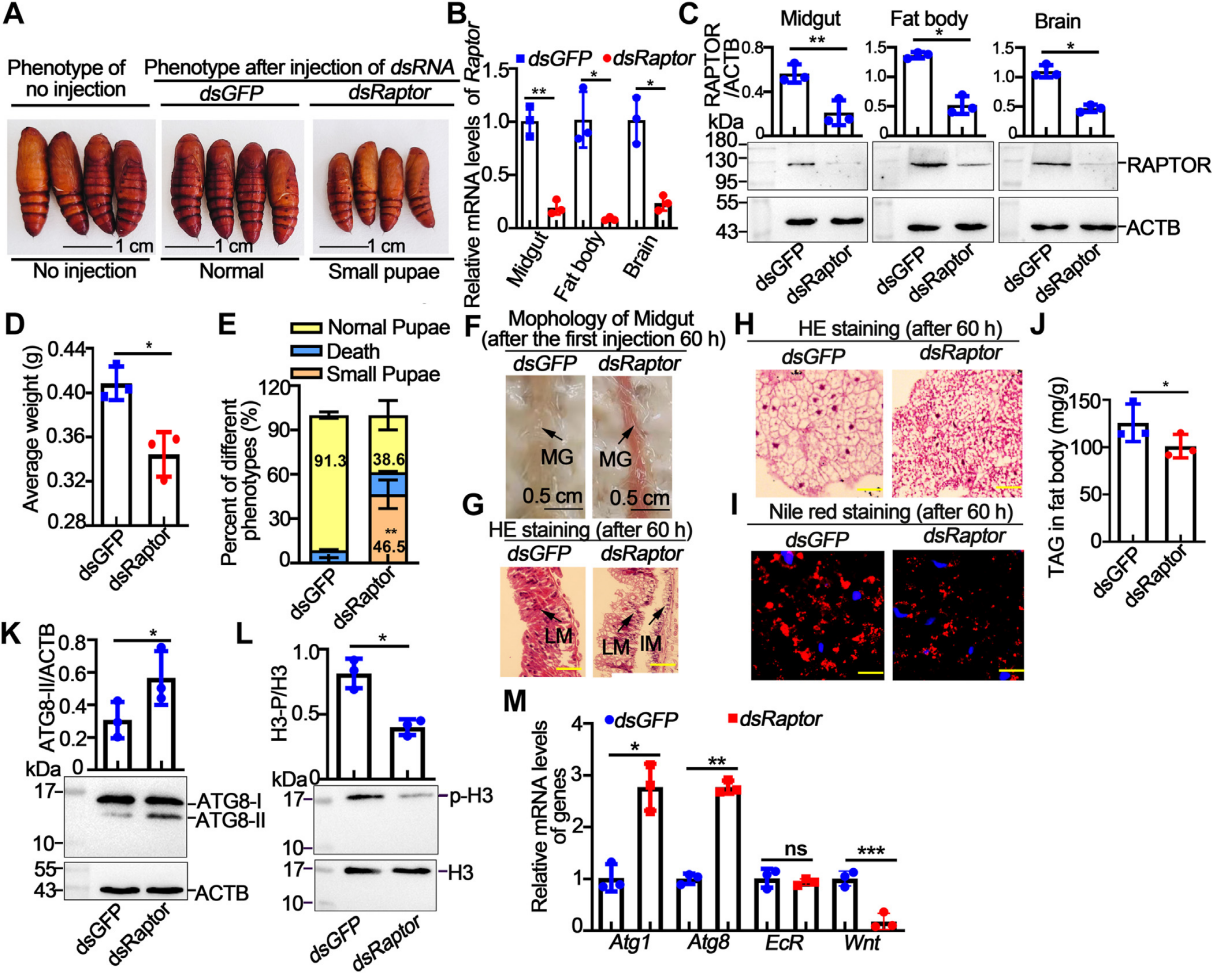
**Knockdown of *Raptor* induced earlier autophagy and led to the formation of small pupae.***A*, phenotypes after *Raptor* knockdown following the injection of *dsRaptor* (2 μg/larva). *B*, quantitative RT–PCR analysis of the efficiency of *Raptor* knockdown. Actin was used as gene control. *C*, interference efficiency of protein levels in larvae after *dsRNA* injection. ACTB was used as a protein quantity control. *D*, statistical analysis of the average pupa weight. *E*, statistical analysis of the percentages of different phenotypes after *dsGFP* or *dsRaptor* injection. *Asterisk* indicates significant differences from *dsGFP* controls. Each group contained 30 larvae. *F*, morphology of the midgut after *dsRNA* injection. *G*, H&E staining of the midgut after *dsRNA* injection. Scale bar of H&E staining represents 20 μm. *H*, H&E staining of the fat body. Scale bar represents 20 μm. *I*, Nile red staining of the fat body after *dsRNA* injection. Nile red fluorescence showed lipid droplets in fat body tissue. *J*, TAG contents in the fat body after *dsRNA* injection. *K*, Western blot and statistical analysis of the expression of ATG8 after dsRNA injection. ACTB was used as a protein quantity control. *L*, Western blot and statistical analysis of p-H3 expression after *dsRNA* injection. Histone H3 was used as a protein quantity control. *M*, quantitative RT‒PCR analysis of gene expression after *dsGFP* and *dsRaptor* injection. Actin was used as gene control. The statistical analysis was performed using Student’s *t* test (two-tailed, ∗*p* < 0.05, ∗∗*p* < 0.01, n = 3). IM, imaginal midgut; LM, larval midgut; p-H3, phosphorylated histone H3; RAPTOR, regulatory-associated protein of TOR; TAG, triglyceride.

To examine the function of RAPTOR in tissues, we injected *Raptor* dsRNA into 6th-6-h-old larvae to knock down *Raptor* expression. The full length of the interfering fragment of *Raptor* is 496 bp (from 76 bp to 569 bp), which contained the conserved Raptor-N-terminal structural domain, and the specific primer sequence is shown in Table S3. The results showed that smaller pupae were produced after knockdown of *Raptor* (Fig. 4*A*). qRT–PCR and Western blot were used to detect the efficiency of RNAi at the mRNA level and protein level (Fig. 4, B and C). This produced approximately 46.5% smaller pupae, with the weight decrease of approximately 0.03 g compared with that of the control group (Fig. 4, D and E). Morphological observations revealed that the larval midgut turned red earlier (Fig. 4*F*), which is characteristic of the degradation that occurs in the larval midgut (31). H&E staining revealed that the imaginal midgut had formed and dissociated from the larval midgut after *Raptor* knockdown (Fig. 4*G*). H&E staining revealed that the fat body was dissociated 60 h after *dsRaptor* injection compared with that in the control group (Fig. 4*H*). The observed phenomena in the midgut and fat body indicated that autophagy occurred prematurely after *dsRaptor* injection. Nile red staining revealed that the lipid droplets were degraded after *dsRaptor* injection (Fig. 4*I*). Triglyceride (TAG) levels were also significantly reduced after *Raptor* was knocked down (Fig. 4*J*). These findings indicate that the fat body had degraded and that lipophagy had occurred. To understand the underlying mechanism, we detected the proliferation and autophagy of the fat body. Western blot analysis revealed that the level of ATG8-II, a marker of autophagy (33), was increased after *dsRaptor* injection (Fig. 4*K*). The level of phosphorylated histone H3 (p-H3), which is an indicator of proliferation, decreased after *Raptor* knockdown (Fig. 4*L*). We further examined the expression profiles of genes related to autophagy and proliferation using qRT‒PCR. Compared with those in the *dsGFP*-injected group, the expression levels of *Atg1* and *Atg8* were increased, and the expression of *Wnt* was decreased. However, the mRNA level of *EcR* (accession number EU526831) did not change significantly (Fig. 4*M*). The cell proliferation signal in the brain decreased significantly after *dsRaptor* injection (Fig. S3). These data suggest that RAPTOR promotes cell proliferation and represses autophagy in larval tissue.

**Figure 5:**
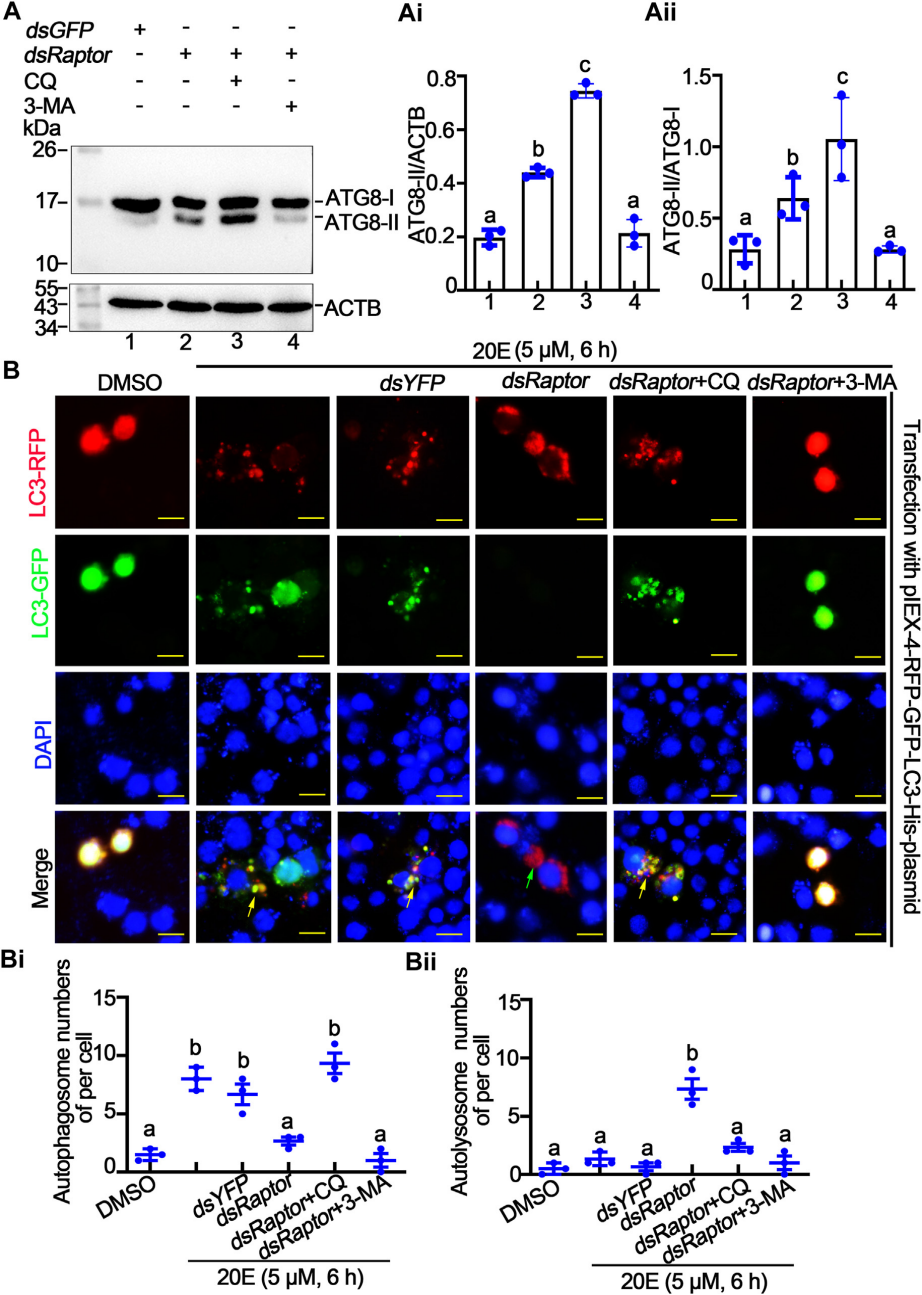
**CQ and 3-MA inhibited autophagy at different stages.***A*, expression of ATG8 in midgut treated with CQ (100 μM) and 3-MA (100 μM) after *dsRNA* injection. *Ai* and *Aii*, statistical analysis of the protein bands in *A*. *B*, autophagy flux detection in HaEpi cell with different treatment. CQ: 50 μM; 3-MA: 50 μM; *dsRNA*: 2 μg. The *yellow arrows* represent autophagosomes, and the *green arrows* represent autolysosomes. Bar represents 20 μm. *Bi* and *Bii*, a count of the puncta number of autophagosome and autolysosome. Statistical analysis was conducted using one-way ANOVA, *different letters* represented significant differences. ATG, autophagy-related gen; CQ, chloroquine; 3-MA, 3-methyladenine.

The autophagy inhibitors chloroquine (CQ) and 3-methyladenine (3-MA) were used to further explore the inhibition of autophagy. The results showed that the amount of ATG8-II and the ratio of ATG8-II to ATG8-Ⅰ were significantly increased after CQ injection in larvae, whereas the amount of ATG8-II and the ratio of ATG8-II to ATG8-Ⅰ were significantly decreased after 3-MA injection (Fig. 5*A*, *A*i and *A*ii). To further explore at which stage of autophagy the two inhibitors inhibit autophagy, we overexpressed pIEx-4-RFP-GFP-LC3-His plasmid in HaEpi cells (established from epidermis of *H. armigera* epidermis in this laboratory) to indicate the autophagy process with different fluorescence. The results showed that autophagosome appeared with 20E treatment for 6 h. The autolysosomes that appeared after *Raptor* knockdown showed quenching of green fluorescence because of the acidic environment of the lysosome. CQ treatment increased the pH of lysosomes and thus disrupted lysosomal function, causing autophagosomes unable to be degrade by the lysosome and GFP unable to be quenched. And 3-MA significantly reduced the number of autophagosome (Fig. 5*B*, *B*i and *B*ii). The aforementioned results indicate that CQ inhibited autophagy at the autolysosome level, whereas 3-MA inhibited autophagy at the initial stage of autophagy.

### RAPTOR promoted adult tissue development

**Figure 6:**
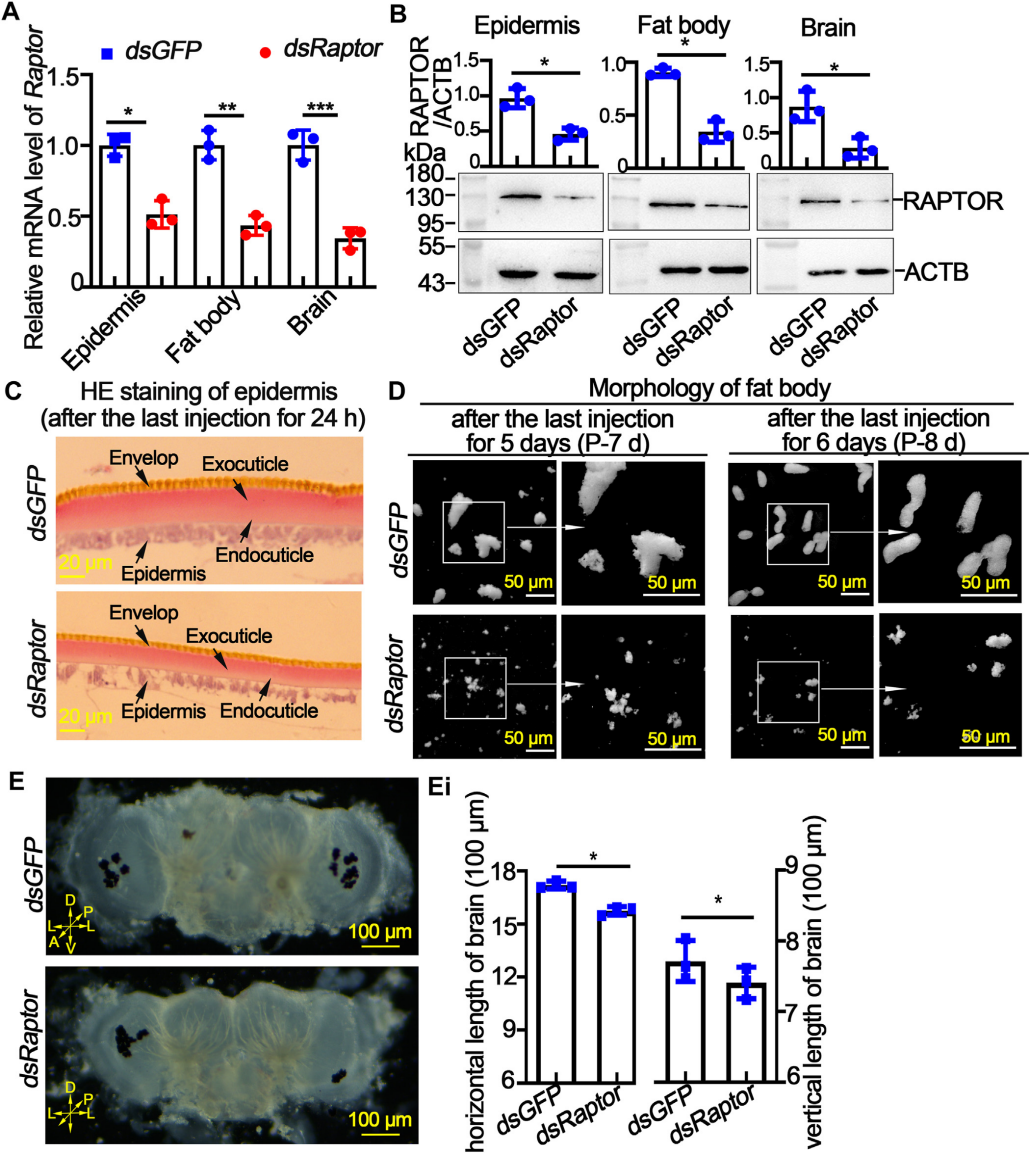
**Knockdown of *Raptor* suppressed imaginal tissue development.***A*, quantitative RT‒PCR analysis of the efficiency of *Raptor* knockdown. Actin was used as gene control. *B*, Western blot showed the efficiency of *Raptor* knockdown in protein level. ACTB was used as a protein quantity control. The statistical analysis was performed using Student’s *t* test (two-tailed, ∗*p* < 0.05, ∗∗*p* < 0.01, ∗∗∗*p* < 0.001, n = 3). *C*, H&E staining of the pupal epidermis after *dsRNA* injection. *D*, morphology of the fat body after *dsRNA* injection. A, anterior; D, dorsal; L, lateral; P, posterior; V, ventral. *E*, brain morphology after *dsRNA* injection. *Ei*, statistical analysis of brain size.

To investigate the function of RAPTOR in imaginal epidermis, fat body, and brain formation, we reduced RAPTOR expression by injecting dsRNA into larvae at 6th–96 h to observe changes in theses tissues in the pupal stage. The interference efficiency on the mRNA level and protein level was verified by qRT‒PCR and Western blot, respectively (Fig. 6, A and B). H&E staining revealed that the outer and inner epidermis thinned after *dsRaptor* injection (Fig. 6*C*). The fat body began to proliferate at P-7 d and formed a complete imaginal fat body at P-8 d in the *dsGFP* control. However, *dsRaptor* injection inhibited the proliferation of the imaginal fat body (Fig. 6*D*). Moreover, the brain size decreased after *Raptor* was knocked down (Fig. 6*E* and *E*i). The aforementioned results indicate that RAPTOR is a key factor for the development of the imaginal epidermis, fat body, and brain.

### HOX proteins promoted *Raptor* transcription

**Figure 7:**
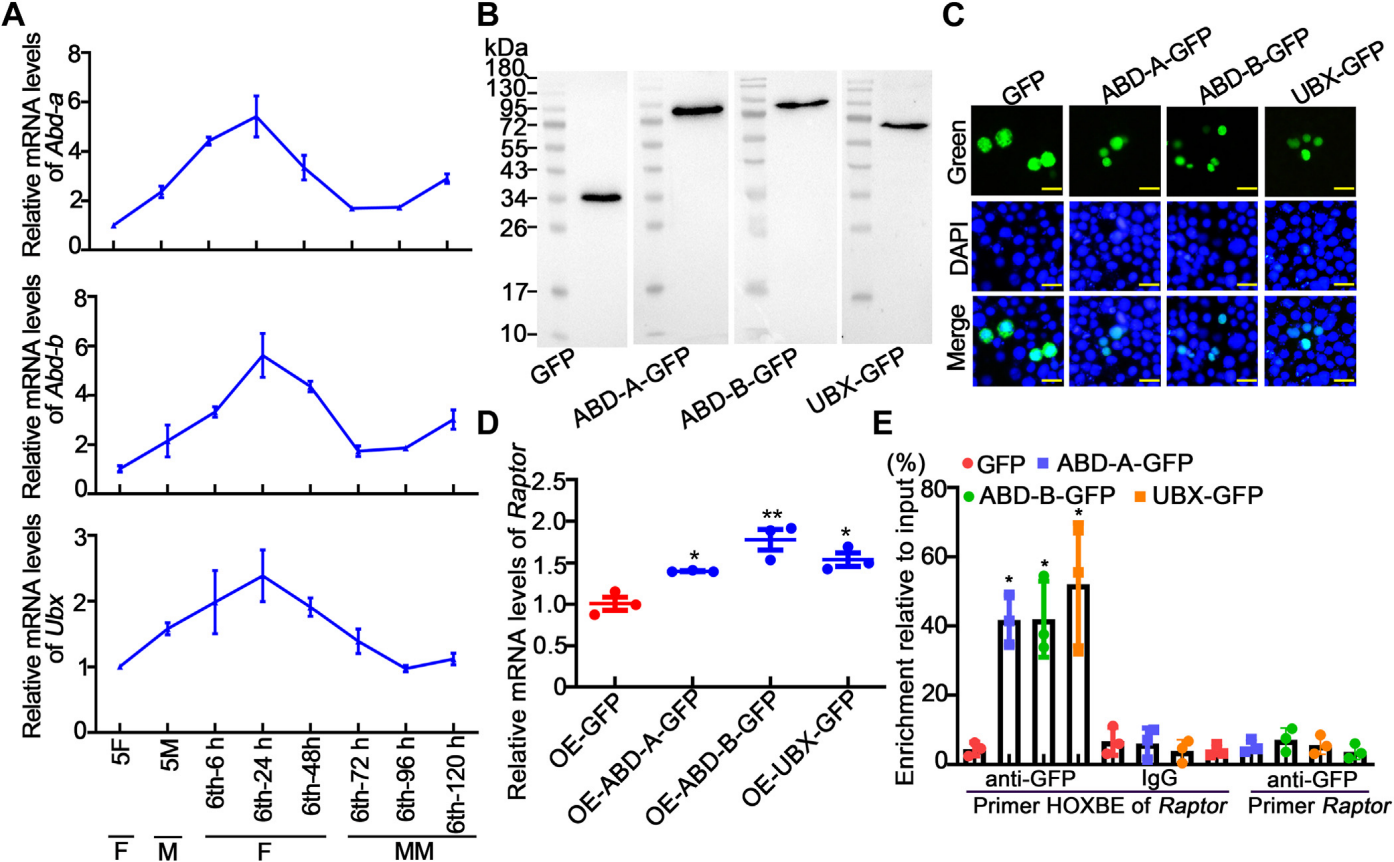
**ABD-A, ABD-B, and UBX promoted *Raptor* transcription.***A*, mRNA levels of *Abd-a*, *Abd-b*, and *Ubx* in the fat body at different developmental stages. Actin was used as gene control. 5F: fifth instar feeding larvae; 5 M: fifth instar molting larvae; 6th-6 h to 6th-6 h–120 h: sixth instar 6 h to 120 h larvae. F, feeding stage; M, molting stage; MM, metamorphic molting stage. *B*, Western blot analysis showing GFP, ABD-A-GFP, ABD-B-GFP, and UBX-GFP levels in HaEpi cells after 72 h of transfection. *C*, the subcellular locations of GFP, ABD-A-GFP, ABD-B-GFP, and UBX-GFP. *Blue*, nuclei stained with DAPI; *green*, GFP, ABD-A-GFP, ABD-B-GFP, and UBX-GFP. Bar represents 20 μm. *D*, *Raptor* mRNA levels in HaEpi cells overexpressing GFP, ABD-A-GFP, ABD-B-GFP, or UBX-GFP. Actin was used as gene control. *E*, ChIP assays showing the binding of ABD-A-GFP, ABD-B-GFP, and UBX-GFP to the HOXBE of *Raptor*. Input: nonimmunoprecipitated chromatin. IgG, nonspecific rabbit IgG. Primer HOXBE of *Raptor*: primers targeting the promoter region containing the HOXBE sequence. Primer *Raptor*: primers targeted to the *Raptor* ORF. The statistical analysis was performed using Student’s *t* test (two-tailed, ∗*p* < 0.05, ∗∗*p* < 0.01, n = 3) and one-way ANOVA (*different letters* represented significant differences). ChIP, chromatin immunoprecipitation; DAPI, 4′,6-diamidino-2-phenylindole; RAPTOR, regulatory-associated protein of TOR.

To identify the transcription factor that promotes *Raptor* expression, we analyzed transcription factor–binding sites using JASPAR (http://jaspar.genereg.net/). The HOX binding element 5′-TTAATTA-3′ (−1097–1103 bp, relative to ATG) is highly conserved with the core sequence 5′-TAAT-3′ (34). Three HOXs, ABD-A, ABD-B, and UBX, have been reported to exist in the fat body of *Drosophila* (35); thus, these three transcription factors were the subjects of our study. The mRNA expression levels of the three *Hox* genes were examined at different developmental stages of the fat body by qRT‒PCR, and the results revealed that *Abd-a*, *Abd-b*, and *Ubx* were highly expressed during the feeding stage but were expressed at low levels during the metamorphosis stage (Fig. 7*A*), similar to the expression pattern of *Raptor*. To further explore the transcriptional regulation of *Raptor* by HOX proteins, we overexpressed three HOXs fused with GFP tags in HaEpi cell lines (Fig. 7*B*), which were established in *H. armigera*, instead of *via* RNAi because of their redundant roles (21). These proteins were all localized in the nucleus (Fig. 7*C*). Compared with the control, overexpression of ABD-A-GFP, ABD-B-GFP, or UBX-GFP significantly increased the expression of *Raptor* (Fig. 7*D*). Chromatin immunoprecipitation (ChIP) assays further demonstrated that ABD-A-GFP, ABD-B-GFP, and UBX-GFP bind more HOXBE-containing DNA sequences than control GFP does (Fig. 7*E*). These results indicated that ABD-A, ABD-B, and UBX promote *Raptor* transcription by binding to HOXBE sequences in the *Raptor* promoter region.

**Figure 8:**
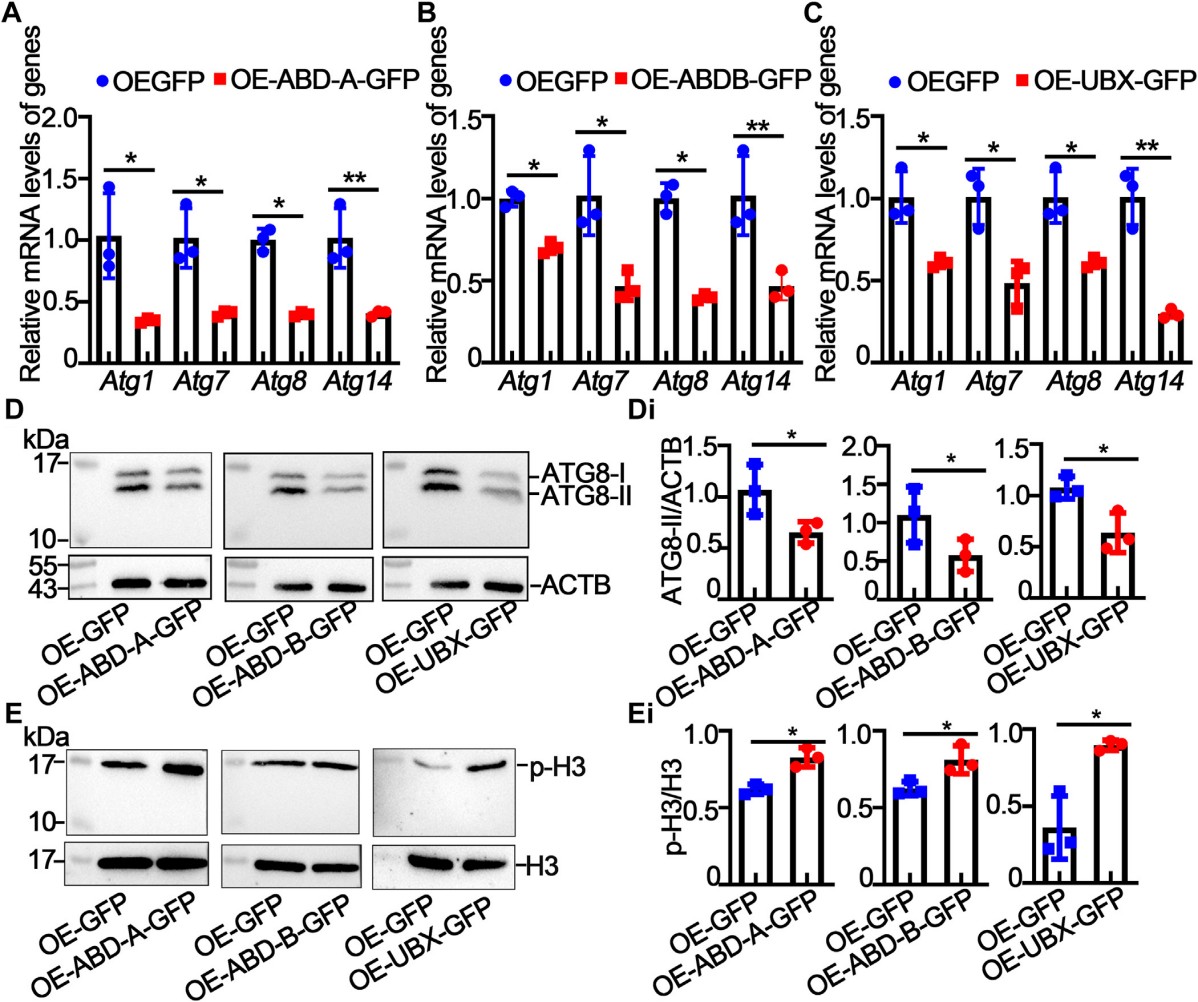
**HOX proteins inhibited cell autophagy and promoted cell proliferation.***A*–*C*, detection of the mRNA levels of autophagy-related genes after the overexpression of GFP, ABD-A-GFP, ABD-B-GFP, and UBX-GFP. Actin was used as gene control. *D*, detection of ATG8 protein levels after overexpression of HOX proteins. ACTB was used as a protein quantity control. *Di*, quantification of ATG8-Ⅱ in *D*. *E*, detection of p-H3 levels after overexpression of HOX proteins. Histone H3 was used as a protein quantity control. *Ei*, quantification of p-H3 in *E*. The statistical analysis was performed using Student’s *t* test (two-tailed, ∗*p* < 0.05, ∗∗*p* < 0.01, n = 3). HOX, homeobox; p-H3, phosphorylated histone H3.

To further explore the function of the transcription factor HOX, autophagy and cell proliferation were examined. Compared with the overexpression of GFP, the overexpression of ABD-A-GFP, ABD-B-GFP, or UBX-GFP promoted the expression of ATGs (Fig. 8, A–C). Western blot analysis revealed that overexpression of ABD-A-GFP, ABD-B-GFP, or UBX-GFP significantly inhibited ATG8 expression (Fig. 8*D* and *D*i). The overexpression of ABD-A-GFP, ABD-B-GFP, or UBX-GFP increased the expression of phosphorylated histones (Fig. 8*E* and *E*i). These results indicate that ABD-A, ABD-B, and UBX promote cell proliferation and inhibit autophagy.

### 20E repressed RAPTOR expression by repressing *Hox* gene transcription

**Figure 9:**
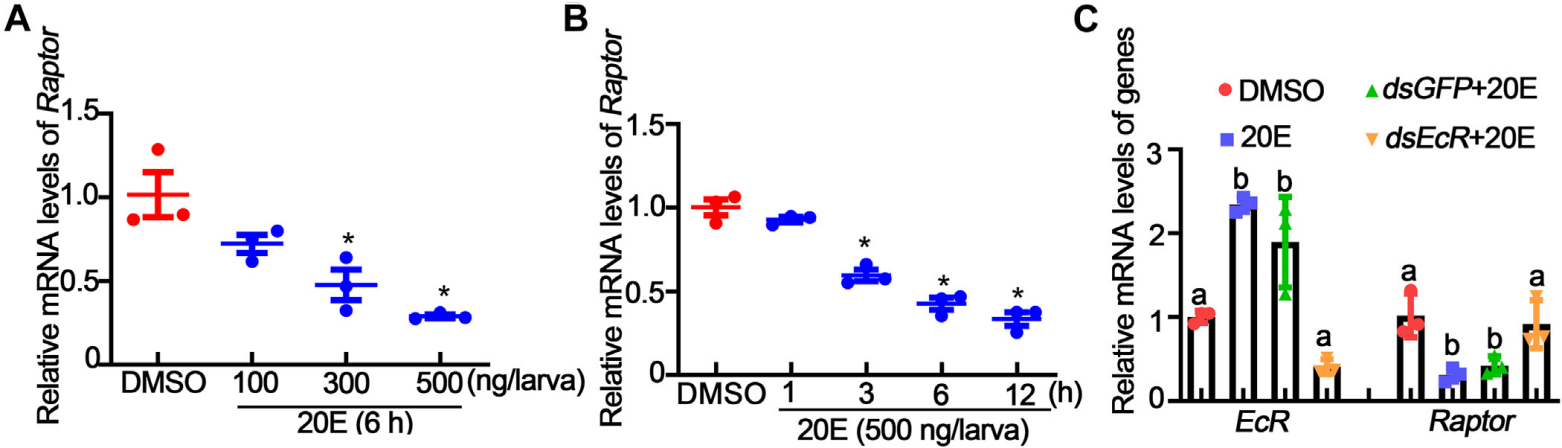
**20E suppressed *Raptor* expression.***A*, *Raptor* mRNA levels in the fat body 6 h after 20E was injected into sixth instars. An equal volume of diluted DMSO (0–500 ng) was used as the solvent control. *B*, *Raptor* mRNA levels in the fat body 1 to 12 h after 20E was injected into sixth-instar larvae at 6 h. *C*, *Raptor* mRNA levels in the fat body after *dsEcR* injection. All the aforementioned data use actin as a gene control. The statistical analysis was performed using Student’s *t* test (two-tailed, ∗*p* < 0.05, n = 3) and one-way ANOVA (*different letters* represented significant differences). 20E, 20-hydroxecdysone; DMSO, dimethyl sulfoxide; RAPTOR, regulatory-associated protein of TOR.

Because RAPTOR expression was significantly decreased in the midgut and fat body during metamorphosis, whereas 20E titers were elevated during metamorphosis, as reported in the literature, thus 20E is likely a suppressor of RAPTOR expression. To test this hypothesis, we injected different concentrations of 20E into the 6th-instar 6 h larval hemocoel. Compared with that in dimethyl sulfoxide (DMSO) treatment, *Raptor* expression was significantly downregulated by 20E in a dose- and time-dependent manner in the fat body (Fig. 9, A and B), suggesting that 20E repressed *Raptor* expression. Furthermore, there was no significant decrease in *Raptor* expression compared with control after knockdown of *EcR*, the nuclear receptor of 20E (Fig. 9*C*). All these data indicate that 20E represses *Raptor* expression.

**Figure 10:**
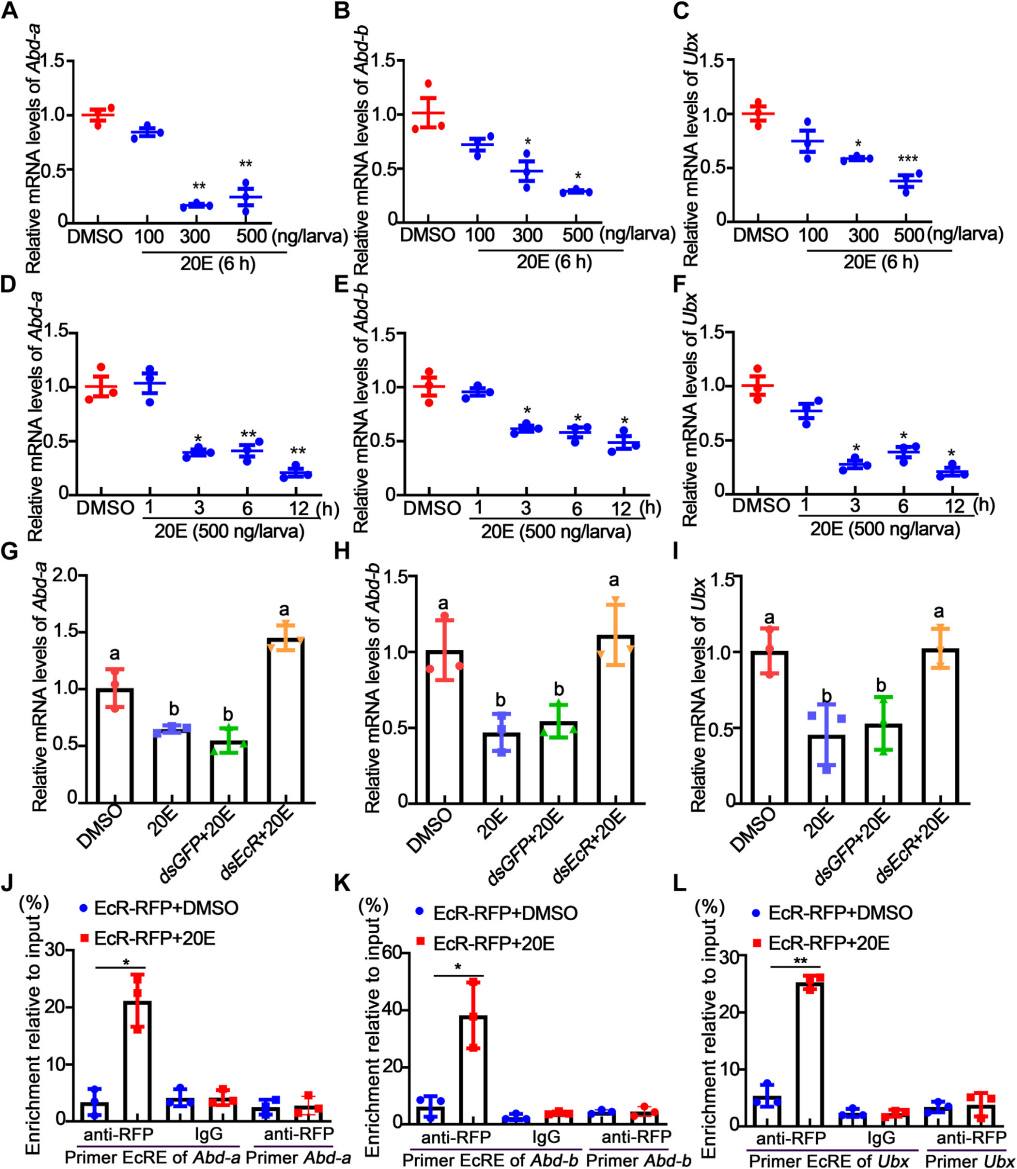
**20E decreased the expression of *Abd-a*, *Abd-b*, and *Ubx via* the EcR.***A*–*C*, mRNA levels of *Abd-a*, *Abd-b*, and *Ubx* in the fat body after injection with 100‒500 ng of 20E for 6 h. *D*–*F*, mRNA levels of *Abd-a*, *Abd-b*, and *Ubx* in the fat body after 500 ng *of* 20E were injected into the sixth instar larvae for 1‒12 h. *G*–*I*, two micrograms of *dsEcR* were injected into the sixth instar larvae, then injected 20E (500 ng/larva) for 12 h to detect the mRNA levels of *Abd-a*, *Abd-b*, and *Ubx* in the fat body. An equal amount of diluted DMSO was used as a control. Statistical analysis of *A–C* was conducted by ANOVA, and *different letters* represent significant differences. Actin was used as a gene control in *A*–*I*. *J*–*L*, ChIP assays revealed that 20E repressed *Abd-a*, *Abd-b,* and *Ubx* transcription *via* the EcR. Input: nonimmunoprecipitated chromatin. Primer EcRE was the sequence containing EcRE. Primers *Abd-a*, *Abd-b*, and *Ubx* were targeted to the *Abd-a*, *Abd-b*, and *Ubx* ORFs. The statistical analysis was performed using Student’s *t* test (two-tailed, ∗*p* < 0.05, ∗∗*p* < 0.01, ∗∗∗*p* < 0.001, n = 3). ChIP, chromatin immunoprecipitation; DMSO, dimethyl sulfoxide.

Similarly, the expression levels of *Abd-a*, *Abd-b*, and *Ubx* were reduced during metamorphosis, so we speculated that 20E also inhibited their expression. We performed hormone concentration gradient and time gradient stimulation experiments to detect the mRNA levels of *Hox* in fat body stimulated with 20E. The expression levels of *Abd-a*, *Abd-b*, and *Ubx* were significantly reduced after 300 to 500 ng 20E treatment for 6 h (Fig. 10, A–C). The expression levels of *Abd-a*, *Abd-b*, and *Ubx* were also significantly reduced after 3 to 12 h of 500 ng 20E treatment (Fig. 10, D–F). These results indicate that 20E inhibits *Hox* expression, which is consistent with the response of *Raptor* to 20E.

To further investigate the molecular mechanism by which 20E represses *Hox* expression, *EcR* knockdown was followed by superimposed 20E treatment to detect whether 20E represses *Hox* expression through EcR. The results showed no significant decrease in the expression of *Abd-a*, *Abd-b*, and *Ubx* after knockdown of EcR compared with control (Fig. 10, G–I). The conserved EcRE was predicted in the promoter regions of three *Hox* genes using JASPAR (http://jaspar.genereg.net/), namely, *Abd-a* (5′-ATAATGC-3′), *Abd-b* (5′-TCATTGA-3′), and *Ubx* (5′-TTGATGA-3′), which are similar to the EcR-binding elements of the *Hr3* promoter in *H. armigera*. We then performed a ChIP assay to verify whether EcR directly inhibited *Hox* expression. The results showed that EcR-RFP bound more EcRE-containing DNA fragments upon 20E stimulation than did DMSO (Fig. 10, J–L). These results suggested that 20E represses *Hox* transcription through the binding of its nuclear receptor EcR at the promoters of *Hox* genes.

## Discussion

In this study, we revealed that RAPTOR promotes cell proliferation and inhibits cell autophagy in both larval and imaginal tissues. HOX proteins are the transcription factors of *Raptor*, which, *via* RAPTOR, promote cell growth and prevent autophagy. 20E, *via* its nuclear receptor/transcription factor EcR, blocks *Hox* transcription, therefore repressing *Raptor* transcription to inhibit cell proliferation and result in autophagy. The regulatory mechanisms of 20E inhibition of *Raptor* expression present an alternative molecular mechanism for autophagy induced by 20E during insect metamorphosis.

### RAPTOR promoted cell proliferation and inhibited autophagy during insect development

RAPTOR contains a highly conserved N-terminal Raptor structural domain as well as terminal WD40 repeat sequences (36). Phylogenetic tree analysis of RAPTOR from *H. armigera* with RAPTOR proteins from other species revealed that RAPTOR is evolutionarily conserved in animal evolution. The function of TORC1, which is regulated by RAPTOR, is also conserved from yeast to eukaryotes (37).

RAPTOR is a core component of mTORC1 and is responsible for anchoring mTOCR1 to the lysosomal membrane and recognizing and binding substrates of TOR, such as S6K, which are then phosphorylated by TOR to promote protein synthesis and cell growth (38). In mammals, reduced RAPTOR expression during nutrient enrichment decreases S6K phosphorylation, leading to a smaller cell size (36). Knockdown of *Raptor* in *Drosophila* leads to decreased levels of S6K phosphorylation, which inhibits cell growth and consequently leads to abnormal wing development (39). In this work, we found that the function of RAPTOR in *H. armigera* development is also to promote cell proliferation and insect growth in larval and pupal tissues, suggesting that the function of RAPTOR is evolutionarily conserved.

Another key role of TORC1 is to prevent autophagy (40). The ULK1–ATG13–FIP200 complex binds directly to mTORC1 *via* RAPTOR adaptation, causing ULK1 to be phosphorylated by mTOR and unable to form an autophagy initiation complex, thereby inhibiting autophagy (41). In *D. melanogaster*, *Raptor* knockdown leads to the upregulation of autophagy-related gene expression and induces earlier and intense autophagy in the midgut or fat body (9). The study also revealed that *Raptor* knockdown caused autophagy, which is consistent with the known inhibition of autophagy by RAPTOR, suggesting that the inhibition of autophagy by RAPTOR was also conserved throughout evolution.

### HOX proteins promoted *Raptor* transcription

*Hox* genes are first identified in *D. melanogaster* and encode transcription factors that play roles in organism development (42). HOX proteins have highly conserved homologous motifs that are responsible for binding to DNA at specifically recognized binding sites, thereby regulating the transcription of their target genes (43). Most HOX proteins recognize and bind to -TAATTG- or -TAATTA-containing sequences to regulate gene expression (44). Here, we found that the BX-C members *Abd-a*, *Abd-b*, and *Ubx* were highly expressed in larval tissues during the feeding stages and that their expression decreased during metamorphosis. The HOX binding element is conserved in the *Raptor* promoter region of *H. armigera*, and HOX proteins promote *Raptor* transcription to repress autophagy during larval growth, which reveals the molecular mechanism of *Raptor* transcriptional regulation and provides an upstream target for regulating *Raptor* expression.

### 20E repressed the expression of *Hox via* the EcR

In mammals, estrogen plays an important role in regulating the transcription of *Hox* genes. In breast cancer cells, estrogen can promote *Hox* transcription through its receptor α (estrogen receptor, ERα) and receptor β (estrogen receptor, ERβ) binding to estrogen response elements (EREs) in the HOXC10 promoter, thereby promoting tumor growth (18). It has also been reported that estrogen can inhibit tumor growth by binding to the ERE in the *HoxB2* promoter region through its receptor ERα (45). In breast cancer cells, overexpression of the estrogen receptor ERα inhibits HOXB7 expression (19), but the specific molecular mechanism has not been elucidated. The growth and development of holometabolous insects are regulated by various hormones, in which the titer of the steroid hormone 20E increases greatly during metamorphosis, and 20E functions as the key hormone to repress growth and promote autophagy (46). EcR is a transcription factor and the nuclear receptor of 20E, which binds to the EcRE in the promoter of the target gene to promote the transcription of genes such as *HR3*, and thus, programmed cell death (47). In *Drosophila*, 20E represses *Hox* expression by repressing Pontin, a member of the chromatin remodeling complex, but the specific transcriptional regulatory mechanism has not been elucidated (21). In this study, we found that EcR can bind to the promoter regions of the *Hox* genes *Abd-a*, *Abd-b*, and *Ubx* to repress their transcription, thereby causing autophagy. This is similar to the role of estrogen in inhibiting the transcription of *HoxB2* through the binding of ERα to the ERE in its promoter region, which plays a role in suppressing tumor growth, and it is speculated that this is the molecular mechanism by which steroid hormones inhibit the transcription of *Hox* genes.

## Conclusion

**Figure 11:**
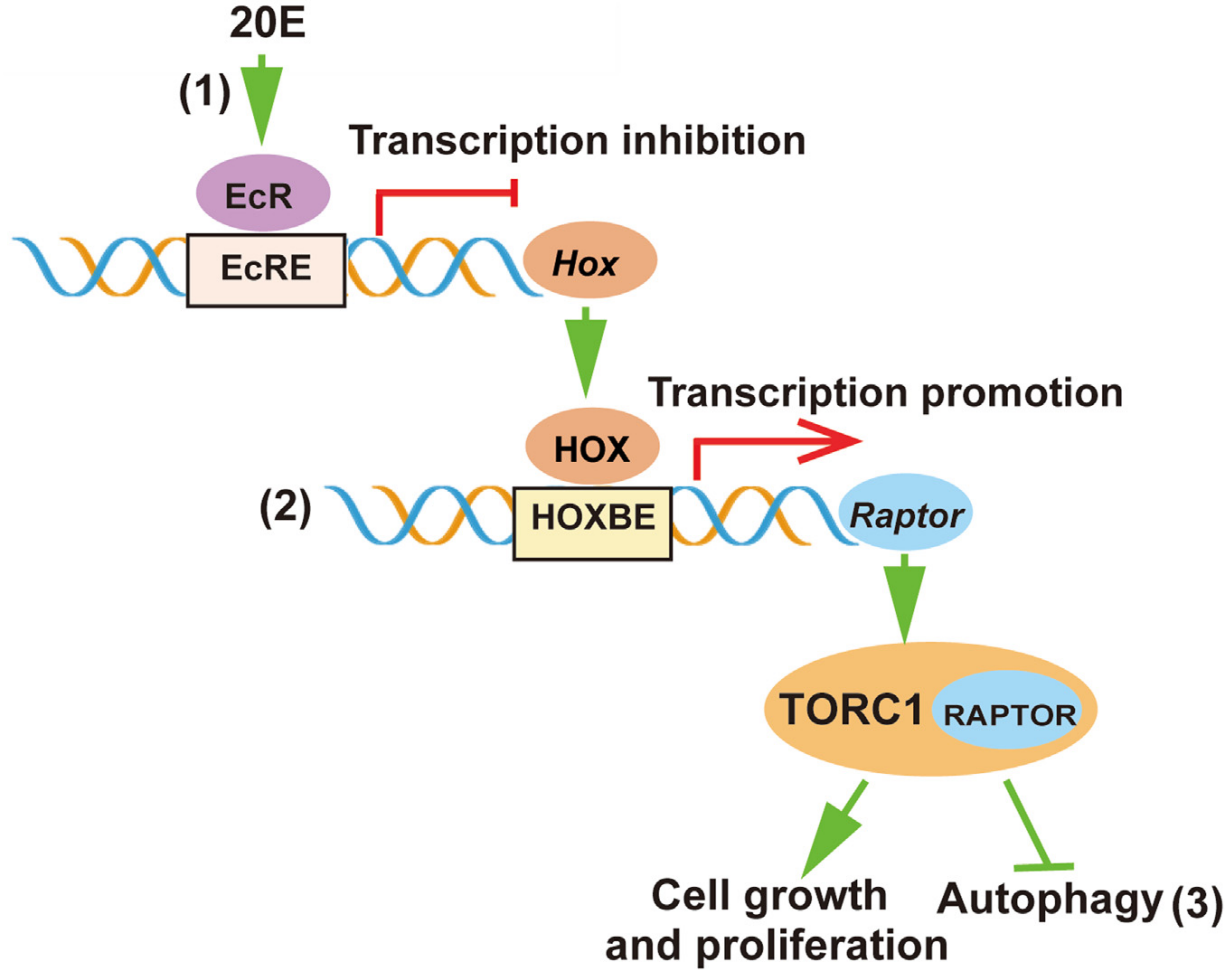
**Diagram showing the mechanism by which 20E represses cell proliferation and promotes autophagy by reducing *Raptor* expression through the inhibition of *Hox* transcription.** 20E represses transcription of the *Hox* gene through its nuclear receptor EcR (1). HOX proteins promote the transcription of *Raptor* (2). RAPTOR promotes cell growth and proliferation and inhibits autophagy through TORC1 (3). 20E, 20-hydroxecdysone; HOX, homeobox; RAPTOR, regulatory-associated protein of TOR.

Taken together, HOX proteins are transcription factors that promote *Raptor* transcription. 20E inhibits *Hox* gene transcription *via* EcR binding to the promoter element of the *Hox* gene, thereby inhibiting *Raptor* expression, which in turn inhibits cell growth and proliferation and promotes cell autophagy (Fig. 11).

## Experimental procedures

### Experimental animals

We purchased *H. armigera* from Keyun Biology on Taobao and cultured them in our laboratory at 27 ± 1 °C with a cycle of 14 h in the dark and 10 h in the light and fed them an artificial diet, which was described previously (48).

### Cell line

The HaEpi cell line was derived from the *H. armigera* epidermis, which was previously well characterized in our laboratory (49). The cells were cultured in cell culture flasks at 27 °C with Grace’s insect cell culture medium supplemented with 10% fetal bovine serum (Gibco) and 2% antibiotics.

### Western blot

Total protein was extracted from the tissue with 40 mM Tris–HCl or radioimmunoprecipitation assay lysis buffer, and the protease inhibitor PMSF was added at a ratio of 1:100 to prevent protein degradation. The supernatant was collected after complete grinding, and the protein concentration was determined by the bicinchoninic acid method. The total cell protein content was also measured in this manner. Next, the protein samples were separated by SDS-PAGE and transferred to nitrocellulose membranes. Antibodies diluted with blocking buffer (5% fat-free powdered milk in 10 mM Tris–HCl, 150 mM NaCl, 0.02% Tween, pH 7.5 was added to the membranes and incubated for 1 h at room temperature. The primary antibodies were diluted with blocking buffer and incubated with the membranes overnight at 4 °C. The dilution of anti-RAPTOR antibodies was 1:1000 (WanLeiBio; WL05043); the dilutions of anti-ACTB (ABclonal Technology; AC026, Research Resource Identifier [RRID]: AB_2768234); anti-H3 (Proteintech; catalog no.: 17168-1-AP; RRID: AB_2716755); anti-p-H3 (Cell Signaling Technology; catalog no.: 9701; RRID: AB_331535); and ATG8 (Abcam,; catalog no.: ab109364; RRID: AB_10861928) were 1:3000. We washed the membranes three times with Tris-buffered saline with Tween-20 and incubated them with a secondary antibody (anti-rabbit IgG coupled to horseradish peroxidase, diluted 1:5000). The bands were detected using a chemiluminescence imaging system.

### Immunohistochemistry

We fixed the midgut with 4% paraformaldehyde for 24 h and then sent the sample to a professional company (Servicebio) for H&E staining and immunofluorescence localization. The preserum and anti-Raptor antibodies were diluted at a ratio of 1:50. The secondary antibody used was Cy3-conjugated goat antirabbit IgG (H+L) (1:200 dilution, GB21303; Servicebio).

### Integral immunohistochemistry of the brain

Intact brain tissue was dissected and obtained under a microscope and fixed in 4% paraformaldehyde for 45 min. The paraformaldehyde was removed, and the samples were washed three times with PBS for 15 min each. Five percent bovine serum albumin was added to PBST (0.3% Triton X-100 in PBS) as a blocking solution. The tissues were added to the blocking solution and blocked for 2 h at room temperature. Anti-RAPTOR antibodies diluted 1:20 with blocking solution were added, and the mixture was incubated at 4 °C for 48 h. The tissue was then washed three times with PBS for 15 min each time. Red fluorescent secondary antibody goat antirabbit IgG secondary antibody DyLight594 diluted 1:1000 with PBS was added, and the samples were incubated for 3 to 4 h at room temperature. This was followed by three 15 min washes with PBS. 4′,6-Diamidino-2-phenylindole diluted in 1:1000 in PBS was added, and the mixture was incubated for 30 min at room temperature. The samples were then washed three times with PBS for 15 min each time. An appropriate amount of blocking agent was added for blocking, and the samples were temporarily stored in a refrigerator at 4 °C to observe the fluorescence signal. Olympus BX51 fluorescence microscope (Olympus Optical Co) was used to observe.

### Hormonal regulation

20E was diluted with DMSO to a concentration of 2 mM. Then, 20E was diluted to a concentration of 100 ng/μl with PBS. Next, 100, 200 or 500 ng of 20E was injected into the hemolymph of 6th-instar 6 h larvae for 6 h, and 500 ng of 20E was injected into the larval hemolymph for 1, 3, 6, 12, or 24 h. An equivalent amount of DMSO was injected as a control. Total mRNA or protein was extracted for qRT‒PCR or Western blotting.

### RNAi in larvae

We diluted the dsRNA to a concentration of 500 ng/μl with aseptic PBS. We then injected 5 μl of dsRNA into 6th-instar larvae at 6 h to observe larval tissue and into 6th-instar larvae at 96 h to observe imaginal tissue. A second injection was given 24 h after the first injection, and the second injection was given 24 h after the first injection. Three injections were performed in total. An equal amount of *dsGFP* was injected as a control. Total RNA or protein was extracted at 24 h after the last injection to measure the efficiency of RNAi.

### TAG determination

A TAG assay kit (BC0620; Solarbio) was used for this assay. Solution I was prepared according to the instructions, and fat bodies from three larvae were removed and ground well in solution I. The supernatant was collected after centrifugation at 800*g* at 4 °C, following the instructions.

### Histone extraction

Fat body tissue was collected, and the steps of the histone extraction kit (BB-3117; Bestbio) were followed. A total of 500 μl of solution A was added, the tissue was ground completely, and the mixture was lysed on ice for 10 min. The precipitate was collected by centrifugation at 16,000*g* for 15 min. Then, 100 μl of solution B was added, and the mixture was mixed thoroughly and incubated at 4 °C overnight. The supernatant was collected by centrifugation at 16,000*g* for 10 min. About 15 μl of solution C and a half volume of protein loading mixture were added, and the mixture was boiled at 100 °C for 10 min.

### qRT‒PCR analysis

We reverse transcribed the RNA into complementary DNA using a reverse transcription kit (G492; Abcam). A 2× SYBR RT‒PCR mixture was used to perform qRT‒PCR, and the primers used are listed in Table S3. The relative expression of the target gene was quantified using *H. armigera* β-actin. Each experiment was repeated at least three times. The 2^−ΔΔCT^ method was used to analyze the data (50).

### Observation of tissue morphology

The epidermis, midgut, and fat body tissues were removed, fixed with 4% paraformaldehyde for 24 h, and then sent to a professional company for H&E staining.

### Autophagy flux detection

HaEpi cells were transferred to a 24-well plate and transfected with 2.5 μg pIEx-4-RFP-GFP-LC3-His plasmid for 48 h. The cells were also transfected with *dsRNA*. 48 h later, and the cells were treated with CQ and 3-MA for 24 h. The red fluorescence was fused with green fluorescence to turn into yellow color when autophagosomes were formed. And when autophagosomes were fused to the lysosome to form autolysosomes, the acid environment in the lysosome quenched the green fluorescence, and only red fluorescence was retained. Therefore, the different fluorescence colors can be used to determine the autophagy process.

### Overexpression in HaEpi cells

A total of 5 μg of plasmid was transfected into HaEpi cells using Quick Shuttle-enhanced Transfection Reagent (KX0110042; Biodragon Immunotech). Approximately 72 h after transfection, we extracted total mRNA for qRT‒PCR. We used an Olympus BX51 fluorescence microscope (Olympus Optical Co) at the appropriate magnification for observation.

### ChIP assay

When the corresponding transcription factors were overexpressed in HaEpi cells, the cells overexpressing EcR-RFP were treated with DMSO or 20E for 6 h. The cells were fixed for 10 min with formaldehyde at a final concentration of 1%, after which glycine was added to terminate crosslinking. The cells were washed three times with PBS after the medium was removed and lysed with SDS lysis buffer. The DNA was sheared by sonication into fragments 100 to 1000 bp in length. After centrifugation, 50 μl of protein A + G was added to the supernatant for 1 h to prevent nonspecific binding. The samples were divided into three aliquots, and one was used as an input, one was used as a control incubated without antibody, and His antibody was added to the third to bind the DNA fragments. Nonspecific mouse IgG was used as a control. The samples were combined at 4 °C overnight. Next, a series of subsequent operations were performed with a ChIP assay kit (Beyotime Biotechnology; P2078). The purified DNA products obtained were tested for enrichment of fragments (containing transcription factor–binding sites) by qRT‒PCR.

### Quantification and statistical analysis

All experiments were repeated at least three times. The data were analyzed using GraphPad 8.0 (GraphPad Software, Inc). Comparisons between two groups were performed with Student’s *t* test (∗*p* < 0.05, ∗∗*p* < 0.01, and ∗∗∗*p* < 0.001), and one-way ANOVA (significant differences are indicated by *different letters*) was performed for multiple comparisons. The error bars represent the means ± SD of three independent experiments. The analysis of protein bands in the Western blot was quantified with ImageJ software (National Institutes of Health, http//imagej.nih.gov/ij/download.html).

### Antibodies

The polyclonal antibodies of anti-RAPTOR were purchased from WanLeiBio (WL05043); the antibody of anti-ACTB was purchased from ABclonal Technology (catalog no.: AC026; RRID: AB_2768234); the antibodies of anti-H3 were purchased from Proteintech (catalog no.: 17168-1-AP; RRID: AB_2716755); the antibodies of anti-p-H3 were purchased from Cell Signaling Technology (catalog no.: 9701; RRID: AB_331535); and the antibodies of ATG8 were purchased from Abcam (catalog no.: ab109364; RRID: AB_10861928). Goat antirabbit IgG secondary antibody DyLight594 was purchased from Abbkine Scientific (catalog no.: A23420).

### Ethics statement

Experiments on the animals concerned were performed in accordance with protocols approved by the Animal Care & Welfare Committee, Shandong University School of Life Sciences (SYDWLL-2021-59).

## Data availability

All data are contained within the article. Any additional information required to reanalyze the data reported in this article is available from the lead contact Xiao-Fan Zhao (xfzhao@sdu.edu.cn) upon request.

## Supporting information

This article contains supporting information.

## Conflict of interest

The authors declare that they have no conflicts of interest regarding the contents of this article.
