# Supplementary material for: The steroid hormone 20-hydroxyecdysone inhibits RAPTOR expression by repressing Hox gene transcription to induce autophagy
Source: J Biol Chem. 2024 Dec 18;301(1):108093. doi: 10.1016/j.jbc.2024.108093 (PMC11786772; doi:10.1016/j.jbc.2024.108093)
Supplement: Supporting information [file mmc1.docx]

**The steroid hormone 20-hydroxyecdysone inhibits RAPTOR expression by repressing *Hox* gene transcription to induce autophagy**

Tian-Wen Liu, Can Tian, Yan-Xue Li, Jin-Xing Wang^*^ and Xiao-Fan Zhao^*^

Shandong Provincial Key Laboratory of Animal Cells and Developmental Biology, School of Life Sciences, Shandong University, Qingdao 266237, China

^*^Corresponding author: Xiao-Fan Zhao, E-mail address: [xfzhao@sdu.edu.cn](mailto:xfzhao@sdu.edu.cn)

Jin-Xing Wang, E-mail address: [jxwang@sdu.edu.cn](mailto:jxwang@sdu.edu.cn)

**Supporting Information：**

Supplemental Figures and Legends: Figure S1-S3

Supplemental Table 1: Table S1-S3


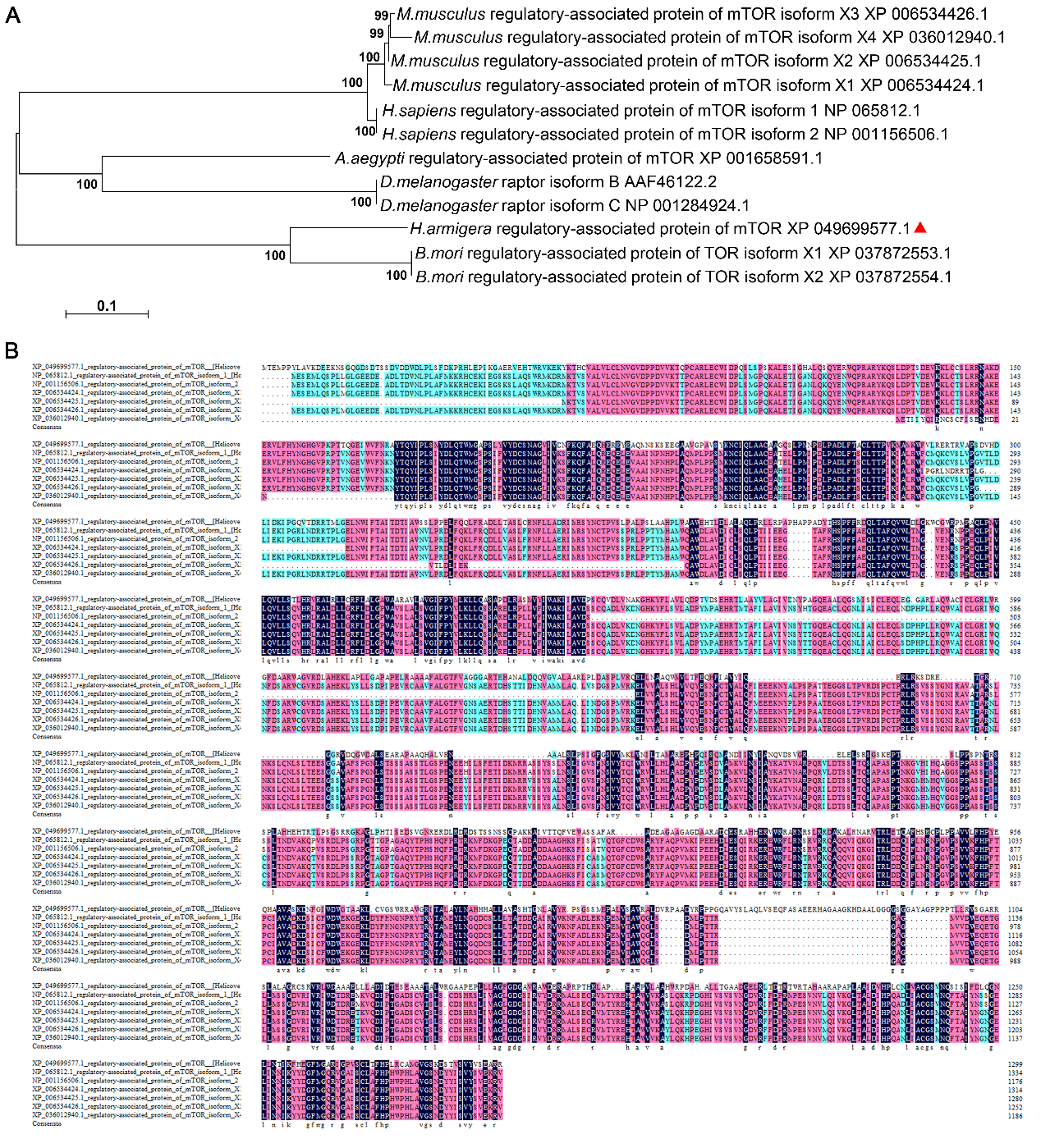


Figure S1. The identification of RAPTOR. A) Phylogenetic trees were constructed for RAPTORs in different species. B) Amino acid sequence alignment of RAPTOR in different species.


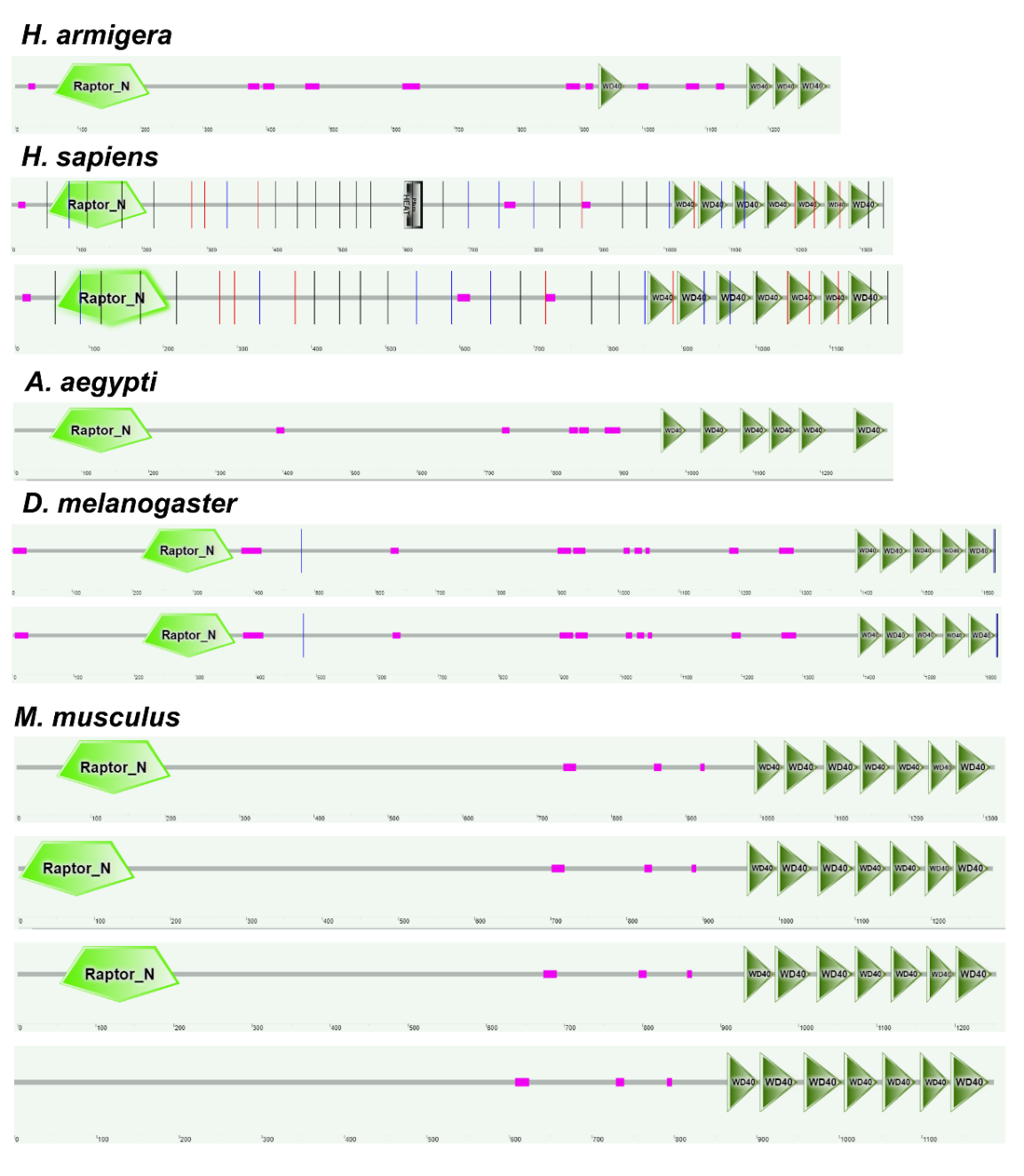


Figure S2. Structural domain analysis of RAPTOR in different species.


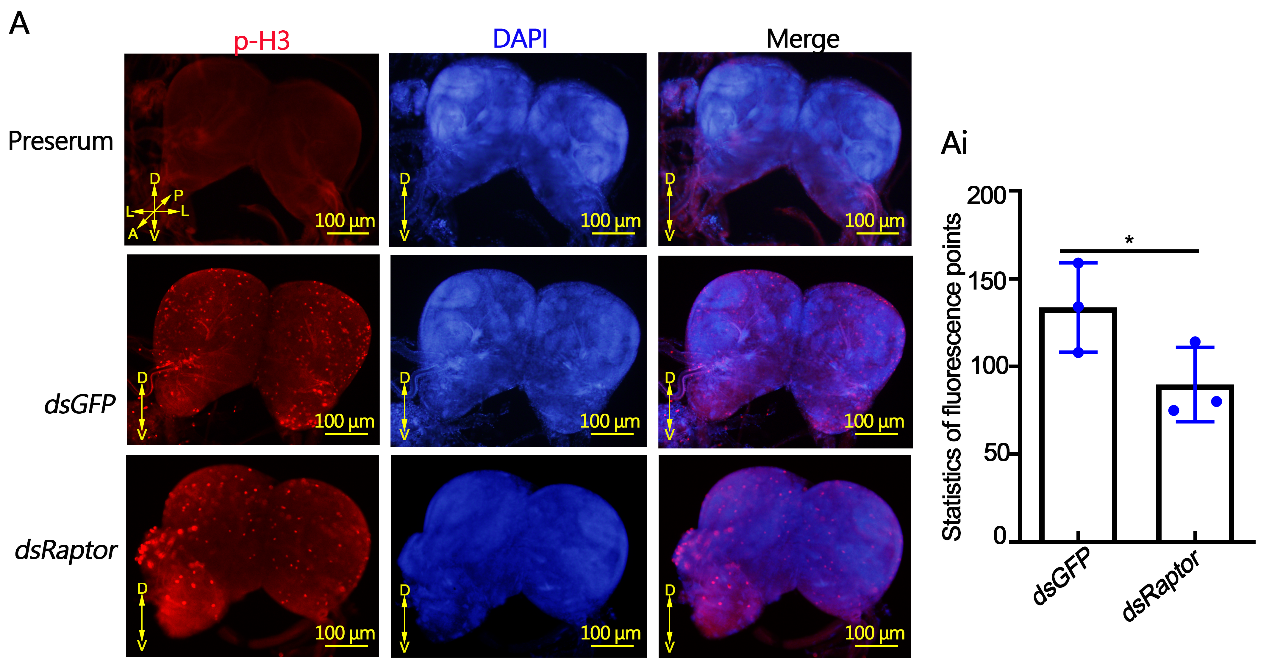


Figure S3. *Raptor* knockdown inhibited proliferation signaling in the brain. *A,* Fluorescence signal of p-H3 in the brain after *dsRaptor* injection. Red, RAPTOR detected using anti-RAPTOR antibodies and goat anti-rabbit IgG secondary antibody DyLight594; blue, nuclei stained using DAPI. A: Anteri, D: Dorsal, L: Lareral, P: Posterior, V: Ventral. *B,* Fluorescence intensity statistics of A.

**Table S1 Statistical analysis of the data in Fig 1A.**

| Fig1A | Epidermis | | | Midgut | | | Fat body | | | Brain | | |
| --- | --- | --- | --- | --- | --- | --- | --- | --- | --- | --- | --- | --- |
| 5F | 0.322985 | 0.216868 | 0.537346 | 0.082272 | 0.402659 | 0.314599 | 1.904778 | 1.696788 | 1.512105 | 1.385544 | 0.222341 | 0.624174 |
| 5M | 0.992701 | 1.05717 | 1.604155 | 1.04238 | 0.98363 | 1.01443 | 0.953172 | 1.273657 | 1.425225 | 0.853495 | 0.324549 | 0.83615 |
| 6th-6 h | 1.119039 | 1.302334 | 1.033724 | 1.071874 | 1.066778 | 1.314165 | 1.245903 | 1.383025 | 1.662485 | 0.827828 | 0.313165 | 0.6895 |
| 6th-24 h | 0.22622 | 0.970094 | 1.065759 | 0.791226 | 1.084467 | 1.107515 | 1.21448 | 1.378763 | 1.798533 | 0.599771 | 0.495839 | 0.685179 |
| 6th-48 h | 0.103868 | 0.533592 | 0.43097 | 0.385246 | 0.270979 | 0.288951 | 1.265591 | 1.198746 | 1.672278 | 0.407537 | 0.318286 | 0.573235 |
| 6th-72 h | 0.15544 | 0.124735 | 0.295739 | 0.257911 | 0.316328 | 0.384056 | 0.86015 | 0.854011 | 1.382177 | 0.811014 | 0.620986 | 0.866846 |
| 6th-96 h | 0.11996 | 0.125863 | 0.384172 | 0.273753 | 0.572032 | 0.824358 | 0.677588 | 0.710183 | 0.999239 | 0.660544 | 0.576511 | 0.933689 |
| 6th-120 h | 0.147495 | 0.633255 | 0.172978 | 0.227127 | 0.567999 | 1.07197 | 0.618774 | 0.653381 | 0.964073 | 0.836334 | 0.56014 | 0.860853 |
| P-2 d | 1.844645 | 1.76758 | 1.234872 | 0.466766 | 0.809268 | 0.549654 | 1.436997 | 1.677513 | 0.764602 | 0.674494 | 0.491115 | 0.110584 |
| P-4 d | 0.673939 | 1.306436 | 0.928409 | 0.185885 | 0.22236 | 0.508406 | 0.770693 | 0.942842 | 0.998092 | 0.89057 | 0.632683 | 0.634441 |
| P-6 d | 1.366802 | 1.049588 | 0.902495 | 0.248676 | 0.120356 | 0.331344 | 0.493547 | 1.165894 | 0.734857 | 1.218063 | 0.406814 | 0.80228 |
| P-8 d | 1.444836 | 1.264578 | 1.889035 | 0.241118 | 0.201663 | 0.325045 | 0.594433 | 1.176402 | 0.280324 | 1.027535 | 0.336549 | 0.553585 |
|  |  |  |  |  |  |  |  |  |  |  |  |  |
| Anova analysis | 5F | 5M | 6th-6 h | 6th-24 h | 6th-48 h | 6th-72 h | 6th-96 h | 6th-120 h | P-2 d | P-4 d | P-6 d | P-8 d |
| Epidermis | a | b | b | b | a | a | a | a | b | b | b | b |
| Midgut | a | b | b | b | a | a | a | a | a | b | a | a |
| Fat body | b | b | b | b | b | a | a | a | a | a | a | a |
| Brain | a | a | a | a | a | a | a | a | a | a | a | a |

**Table S2 Statistical analysis of the data in Fig 1B.**

| Fig1B | Midgut | | | Epidermis | | | Fat body | | | Brain | | |
| --- | --- | --- | --- | --- | --- | --- | --- | --- | --- | --- | --- | --- |
| 5F | 0.732043 | 0.806642 | 0.959264 | 1.054579 | 0.710382 | 1.33484 | 7.447038 | 5.683055 | 7.344512 | 2.042024 | 2.027919 | 2.584706 |
| 5M | 1.025741 | 0.82169 | 0.97041 | 1.443929 | 2.34567 | 1.494849 | 18.29444 | 13.57926 | 11.73978 | 2.874544 | 1.605846 | 1.605846 |
| 6th-6 h | 5.181369 | 4.237852 | 2.757447 | 5.883467 | 5.965598 | 5.489469 | 20.77342 | 16.52601 | 17.71214 | 2.887858 | 1.558329 | 1.892115 |
| 6th-24 h | 7.926405 | 8.917669 | 6.758329 | 4.046478 | 4.713069 | 3.498331 | 10.65407 | 11.82144 | 12.6699 | 2.009263 | 2.276262 | 2.632925 |
| 6th-48 h | 5.775717 | 6.408559 | 5.578975 | 4.648182 | 4.680513 | 4.8121 | 8.633826 | 12.99604 | 9.917662 | 2.383916 | 3.348078 | 3.279176 |
| 6th-72 h | 1.307369 | 1.900879 | 1.107009 | 2.928171 | 2.411616 | 2.34567 | 6.105037 | 7.78124 | 6.773963 | 4.121968 | 3.563595 | 4.326899 |
| 6th-96 h | 1.685683 | 1.54043 | 1.467472 | 2.329467 | 2.143547 | 2.158456 | 8.917669 | 8.092956 | 7.709657 | 3.182146 | 2.907945 | 2.34567 |
| 6th-120 h | 1.41095 | 2.094588 | 1.512219 | 1.914101 | 2.123828 | 2.009263 | 7.012846 | 6.19026 | 7.260153 | 1.887749 | 1.091768 | 2.08012 |
| P-2 d | 1.430646 | 1.798341 | 2.543238 | 3.160165 | 3.138336 | 3.837056 | 11.18376 | 15.92623 | 7.585968 | 5.110036 | 5.476801 | 5.145579 |
| P-4 d | 3.402669 | 5.01645 | 3.286761 | 5.121856 | 8.917669 | 10.31496 | 10.82779 | 10.82779 | 7.447038 | 4.913213 | 5.683055 | 3.723519 |
| P-6 d | 1.154019 | 2.183537 | 1.280464 | 5.762387 | 6.665285 | 6.483023 | 8.693879 | 8.397733 | 11.71269 | 5.540438 | 7.94474 | 6.680703 |
| P-8 d | 1.967913 | 3.379165 | 1.512219 | 4.228072 | 5.133704 | 2.80889 | 7.48153 | 8.301277 | 11.18376 | 7.533568 | 8.018505 | 5.181369 |
|  |  |  |  |  |  |  |  |  |  |  |  |  |
|  | 5F | 5M | 6th-6 h | 6th-24 h | 6th-48 h | 6th-72 h | 6th-96 h | 6th-120 h | P-2 d | P-4 d | P-6 d | P-8 d |
| Epidermis | a | a | b | b | b | a | a | a | a | c | b | a |
| Midgut | a | a | b | c | b | a | a | a | a | b | a | a |
| Fat body | a | b | c | b | b | a | a | a | a | a | a | a |
| Brain | a | a | a | a | a | b | a | a | a | b | a | a |

**Table S3 Primers used in this study**

| **Primer name** | **Sequence (5’-3’)** |
| --- | --- |
| **qRT-PCR** |  |
| *Raptor*-RTF | TAGTGAAGACTCAGCCCTGC |
| *Raptor* -RTR | TTCTTCACCTCATCGCTGGT |
| *Wnt*-RTF | TTGTCAGCAGTAGCCAAGGG |
| *Wnt*-RTR | GCTCGTGATGGCGTAGATGA |
| *Atg1*-RTF | GCGGACCACCTCCATCAA |
| *Atg1*-RTR  *Atg7*-RTF  *Atg7*-RTR  *Atg8*-RTF  *Atg8*-RTR  *Atg14*-RTF  *Atg14-*RTR  *Abd-a* RTF  *Abd-a* RTR  *Abd-b* RTF  *Abd-b* RTR  *Ubx* RTF  *Ubx* RTR  *EcR* RTF  *EcR* RTR | CACTCGCTCCCTGGCTTTC  AAGCCAGCGTGCCTCCCTA  CTGTCTGAATGGCGGGCGA  GAAGAGAAAGACCGAAGGCG  TCAGGTCGTAGATGTATGCGT  ATGGAGATAGAAGAGTTGAGC  CACATGGAGGCGAATGAC  GTCTGCGGTGAGTTCAATGG  GAGCGCGTGTGCTATCTCTA  AAGCAGAAGAGGTGGGAGTTGG  GGTTGTTCGCGTTCGAGTTGT  AAACGGCCTGAGAAGACGAG  CTCCGTCAAACAGAGAGCGT  AATTGCCCGTCAGTACGA  TGAGCTTCTCATTGAGGA |
| *β-Actin* RTF | CCTGGTATTGCTGACCGTATGC |
| *β*-Actin RTR  **Overexpresion**  ABD-A-GFP F  ABD-A-GFP R  ABD-B-GFP F  ABD-B-GFP R  UBX-GFP F  UBX-GFP R  EcR-RFP F  EcR-RFP R | CTGTTGGAAGGTGGAGAGGGAA  TCGTTAACACGTCAAGAGCTCATGAGTTCCAAGTTCATCATCG  ACCGGTACCGTCGACCTGCAGCGTAGGGACCTTGTTCACTTTG  TCGTTAACACGTCAAGAGCTCATGATGAACGGGGTCGGCGGCG  ACCGGTACCGTCGACCTGCAGCTGATGGTGCTTGGGCGGCGGC  TCGTTAACACGTCAAGAGCTCATGAACTCCTATTTCGAGCAGGG  ACCGGTACCGTCGACCTGCAGGTGTTCGGGGTGGCCTTG  Taacacgtcaagagctcatgagacgccgctggtataac  gcaggcgcgccgagatctggagcgccggcgagtccgcca |
| **RNA interference** |  |
| *Raptor* RNAi F | GCGTAATACGACTCACTATAGGAATGCCTCCTTATCTCGCGG |
| *Raptor* RNAi R | GCGTAATACGACTCACTATAGGCCGTGTCCGTTGTAGTGGAA |
| *GFP* RNAi F | GCGTAATACGACTCACTATAGGGATGGTCCCAATTCTCGTGGAAC |
| *GFP* RNAi R  *EcR* RNAi F  *EcR* RNAi R  **ChIP** | GCGTAATACGACTCACTATAGGGACTTGAAGTTGACCTTGATGCC  GAGTAATACGACTCACTATAGGGACGCTGGTATAACAACGGAGGAG GAGTAATACGACTCACTATAGGGAAGCTGGAGCAACTCCTCACG |
| *Raptor* P F  *Raptor* P R  *Aba-a* P F  *Aba-a* P R  *Aba-b* P F  *Aba-b* P R  *Ubx* P F  *Ubx* P R | GTTTCAGGACGCCAGGATAATGA  CCCACTAGACCACCACGACT  ACATGTTTTTATATCTGCAAAAT  TGATTTTTAATGAGGTTAATGAC  GTCCGGGTTTCCAAGTATCTGT  CTTGCACACAATAATGGCGAAA  GCAGATTTCAAAGAATTTATCGTT  TGAGTGCGTGTAATTGGTCAA |
